# Consensus Recommendations for Clinical Outcome Assessments and Registry Development in Ataxias: Ataxia Global Initiative (AGI) Working Group Expert Guidance

**DOI:** 10.1007/s12311-023-01547-z

**Published:** 2023-04-05

**Authors:** Thomas Klockgether, Matthis Synofzik, AGI working group on COAs and Registries, Saud Alhusaini, Mathieu Anheim, Irina Antonijevic, Tee Ashizawa, Luis Bataller, Mélanie Berard, Enrico Bertini, Sylvia Boesch, Pedro Braga-Neto, Emanuel Cassou, Edwin Chan, Rosalind Chuang, Abbie Collins, Joana Damásio, Karina Donis, Antoine Duquette, João Durães, Alexandra Durr, Rebecca Evans, Jennifer Faber, Jennifer Farmer, Vincenzo Gennarino, Holm Graessner, Marcus Grobe-Einsler, Hasmet Hanagasie, Morteza Heidari, Henry Houlden, Elisabetta Indelicato, Kinya Ishikawa, Heike Jacobi, Laura Jardim, Yaz Kisanuki, Svetlana Kopishinskaia, Gilbert L´Italien, Roderick Maas, Michelangelo Mancuso, Caterina Mariotti, Norlinah Mohamed Ibrahim, Wolfgang Nachbauer, Andrea Nemeth, Yi Shiau Ng, Katja Obieglo, Osamu Onodera, Puneet Opal, Luis Pereira de Almeida, Susan Perlman, Guido Primiano, Mathilde Renaud, Liana Rosenthal, Francesco Saccà, Zahid Sattar, Tanja Schmitz-Hübsch, Ludger Schöls, Rebecca Schüle, Lauren Seeberger, Gabriella Silvestri, Anna Sobanska, Bin-Weng Soong, Achal Kumar Srivastava, Colleen Stoyas, Sophie Tezenas du Montcel, Andreas Thieme, Dagmar Timmann, Adina Tocoian, Andreas Traschütz, Bart van de Warrenburg, Wolfram Ziegler

**Affiliations:** 1 Department of Neurology, University Hospital Bonn, Bonn, Germany; 2 German Center for Neurodegenerative Diseases (DZNE), Venusberg-Campus, 53127 Bonn, Germany; 3 Division Translational Genomics of Neurodegenerative Diseases, Center for Neurology, Hertie-Institute for Clinical Brain Research, University of Tübingen, Tübingen, Germany; 4 German Center for Neurodegenerative Diseases (DZNE), Tübingen, Germany

**Keywords:** Activities of daily living (ADL), Ataxia, Clinical outcome assessment (COA), Scale for the assessment and rating of ataxia (SARA), Standardization

## Abstract

To accelerate and facilitate clinical trials, the Ataxia Global Initiative (AGI) was established as a worldwide research platform for trial readiness in ataxias. One of AGI’s major goals is the harmonization and standardization of outcome assessments. Clinical outcome assessments (COAs) that describe or reflect how a patient feels or functions are indispensable for clinical trials, but similarly important for observational studies and in routine patient care. The AGI working group on COAs has defined a set of data including a graded catalog of COAs that are recommended as a standard for future assessment and sharing of clinical data and joint clinical studies. Two datasets were defined: a mandatory dataset (minimal dataset) that can ideally be obtained during a routine clinical consultation and a more demanding extended dataset that is useful for research purposes. In the future, the currently most widely used clinician-reported outcome measure (ClinRO) in ataxia, the scale for the assessment and rating of ataxia (SARA), should be developed into a generally accepted instrument that can be used in upcoming clinical trials. Furthermore, there is an urgent need (i) to obtain more data on ataxia-specific, patient-reported outcome measures (PROs), (ii) to demonstrate and optimize sensitivity to change of many COAs, and (iii) to establish methods and evidence of anchoring change in COAs in patient meaningfulness, e.g., by determining patient-derived minimally meaningful thresholds of change.

Ataxias, which have long been considered untreatable, are now becoming models for the development of targeted therapies. Consequently, an increasing number of observational studies and clinical trials is expected within the next years [1]. This situation requires harmonization and consensus on the use of appropriate clinical outcome assessments (COAs). In this manuscript, we provide (1) an overview of available COAs and (2) a proposal for the data content of ataxia registries based on the consensus of the Ataxia Global Initiative (AGI) working group on COAs.

## Types of Clinical Outcome Assessments (COAs)

According to a draft guidance of the US Food and Drug Association (FDA) published in 2022, a COA is a measure that describes or reflects how a patient feels or functions (https://www.fda.gov/regulatory-information/search-fda-guidance-documents/patient-focused-drug-development-selecting-developing-or-modifying-fit-purpose-clinical-outcome). Validated COAs are indispensable for clinical trials, but similarly important for observational studies in particular natural history studies that serve to prepare these trials. In addition, COAs are also useful in routine patient care.

There are different types of COAs, which complement each other (https://www.fda.gov/regulatory-information/search-fda-guidance-documents/patient-focused-drug-development-selecting-developing-or-modifying-fit-purpose-clinical-outcome):

- Patient-reported outcome measures (PROs) come directly from the patient, without amendment or interpretation of the patient’s response by a clinician or anyone else, and are useful for the patient self-assessment of symptoms, such as unsteadiness or trouble speaking clearly.
- Observer-reported outcome measures (ObsROs) are based on a report of observable signs, events, or behaviors related to a patient’s health condition by someone other than the patient or a health professional, e.g., a parent or caregiver. They are particularly useful for studies of children or cognitively impaired people. While there are, to our knowledge, no ataxia-specific ObsROs, application of activities of daily living (ADL) or patient symptoms score based on parents’ report of an ataxic child would qualify as ObsRO use.
- Clinician-reported outcome measures (ClinROs) come from a trained health-care professional using clinical judgment or interpretation of observable signs, behaviors, or other manifestations related to a disease or condition. In contrast to PROs, which are related to symptoms, ClinROs assess observable signs, such as dysmetria.
- Performance outcome measures (PerfOs) are based on standardized tasks actively undertaken by a patient according to a set of instructions. Performance is usually described by quantitative measures.
- Disease staging is a clinically based measure of severity that uses objective medical criteria to assess the stage of disease progression [2].

## Ataxia-Specific COAs

There are a number of detailed review articles on the properties of ataxia-specific COAs [3–7]. In this paragraph, we therefore limit ourselves to a brief introduction and characterization of the available ataxia-specific COAs and staging systems. This overview is partly based on our previous recommendations on a standardized assessment of ataxia patients in clinical studies [4].

### Patient-Reported Outcome Measures (PROs)

Part II of Friedreich’s ataxia rating scale (FARS) assesses ADL. It consists of nine items. Although FARS-ADL was specifically developed for Friedreichs’s ataxia (FRDA), it is also frequently used for other ataxias [8]. It is primarily designed as a ClinRO, as in its original version it is phrased in the third person and partly includes amendments and interpretations of the clinician, e.g., in the EFACTS study, where it is completed by the clinician as part of an interview with the patient [9]. However, some ataxia networks have used it partly as a PRO, with the patient completing the score fully on his/her own.

Recently, the patient-reported outcome measure of ataxia (PROM-Ataxia) has been introduced. PROM-ataxia has 70 items emerging from patient experience, which are grouped in three domains: physical, ADL, and mental. PROM-ataxia was developed using online surveys in a large group of ataxia patients. The instrument meets generally accepted criteria for reliability, responsiveness to ataxia severity, internal consistency, and item–total score correlations. For validation, it was tested against measures of ataxia and quality of life [10]. However, longitudinal data demonstrating sensitivity to change are currently missing. Additional longitudinal data are currently being acquired to further test its sensitivity to change.

### Clinician-Reported Outcome Measures (ClinROs)

The International Cooperative Ataxia Rating Scale (ICARS) was the first ataxia scale. ICARS has been widely used in observational studies as well as in interventional trials. It consists of 19 items grouped into four subscales that contribute to a total score of 100 points. Subdivisions of different ataxia components are postural and gait disturbance, limb ataxia, dysarthria, and oculomotor disorders [11]. There are two modified versions of the ICARS: the Brief Ataxia Rating Scale (BARS) for use by movement disorder specialists and general neurologists and the modified ICARS (MICARS), in which further items were added [12].

The SARA is currently the most widely used ataxia scale [13, 14]. SARA is based on a semiquantitative assessment of cerebellar ataxia. It has eight unequally weighted items evaluating to gait, stance, sitting, speech, finger‐chase test, nose‐finger test, fast alternating movements, and heel‐shin test. Oculomotor functions are not included in the SARA [15]. In a 1-year follow-up study of 171 patients with SCA1, SCA2, SCA3, or SCA6, SARA was sensitive to change with moderate effect size [16]. In a phase III clinical trial in spinocerebellar ataxia (SCA) patients (https://clinicaltrials.gov/ct2/show/NCT03701399), a shortened and modified version of SARA, named Modified Functional SARA (f-SARA), was used. Another modification is SARA^home^, a video-based instrument, for measuring ataxia severity easily and independently at home [17]. A thorough analysis of the metric properties of these modified versions of the SARA and their degree of sensitivity to longitudinal change, in particular, compared to the original SARA, has not been done [1].

While ICARS and SARA were devised for use in ataxia in general, part III of FARS and the Neurological Examination Score for Spinocerebellar Ataxia (NESSCA) have been devised for specific ataxia diseases, namely Friedreich’s ataxia (FRDA) and spinocerebellar ataxia type 3 (SCA3), respectively [18, 19]. Both ClinROs assess not only ataxia, but also take into account non-ataxia signs that occur in these diseases. Part III of FARS comprises bulbar, upper and lower limbs, peripheral nerve, upright stability, and gait functions. In a modified FARS version (mFARS) used in interventional trials, the number of bulbar items was reduced, and peripheral items were omitted [20]. Due to this, the domain structure of mFARS resembles that of general ataxia ClinROs, with substantial overlap in particular to the SARA. In addition to ataxia, NESSCA assesses a number of non-ataxia signs, such as pyramidal signs, eyelid retraction, ophthalmoparesis, fasciculations, sensory loss, and basal ganglia signs [18].

As various non-ataxic neurological signs may accompany ataxia not only in FRDA and SCA3, but also in a wide range of other ataxias, an additional use of a ClinRO that assesses non-ataxia signs may be useful. For this purpose, the Inventory of Non-Ataxia Signs (INAS) was developed and validated. INAS comprises a list of various non-ataxia signs that often occur in ataxia patients and of cerebellar oculomotor signs that are not considered in the SARA. INAS has 30 items related to neurological signs, such as spasticity, and to reported abnormalities, such as dysphagia. As a simple quantitative measure, the INAS count reflects the severity of non-ataxia involvement [21].

As a consequence of cerebellar dysfunction, ataxia patients may have a syndrome of impaired executive functions, visuospatial cognition, linguistic functions, and personality changes, named the Cerebellar Cognitive Affective Syndrome (CCAS) [22]. Consideration of the CCAS in clinical routine is facilitated by the availability of a validated bedside test, the CCAS scale [23], albeit showing limitations in its application for individual diagnostics [24].

### Performance Outcome Measures (PerfOs)

For ataxia, a number of tests are in use that measure performance in specific coordinative tasks in a quantitative fashion. The Friedreich Ataxia Functional Composite (FAFC) was specifically designed for FRDA. It is derived from the Multiple Sclerosis Functional Composite (MSFC) and composed of a timed 25-ft walk, the 9-hole pegboard test (9HPT), and low-contrast visual acuity (LCVA) [25]. The SCA Functional Index (SCAFI) is very similar to the FAFC, but LCVA was replaced by a speech test (PATA repetition rate). All components are transformed to parameters of performance speed to describe ataxia severity. The SCAFI score is the arithmetic mean of the Z scores of the three tests in relation to a reference cohort. Due to this, the comparison of SCAFI scores between studies is difficult [26]. The Composite Cerebellar Functional Severity Score (CCFS) combines two tests of upper limb function: the 9HPT and the click test [27]. Measurements are adjusted for age and can be compared across different studies. Although ataxia-specific PerfOs yield quantitative and objective data, they were less sensitive to change than ClinROs [28, 29].

### Ataxia Stages

For staging of ataxias, a system including four stages related to walking ability and a final stage defined by death is in use. Information is retrieved by interview of patients, relatives, and caregivers and from medical records [30]. FARS part I (functional staging) defines a finer-graded system of seven functional stages for ataxia. Increments of 0.5 may be used if the status is between stages. As stage 1.0 is defined as “minimal signs detected by physician during screening,” it requires a clinical examination [19]. Both systems correspond to the definition of disease staging given above [2].

## Consensus Recommendations

To accelerate and facilitate the conduct of clinical trials on a global scale, the Ataxia Global Initiative (AGI) was recently established as a worldwide research platform for the trial readiness of ataxias [1]. Both planning and execution of trials are challenged by large between- and within-center variability in outcome assessment [17, 31]. This includes literally all outcome domains, e.g., variability in clinical, digital, imaging, and molecular outcome assessment. To overcome this challenge, the AGI has established working groups that have developed consensus on cross-center, harmonized standard operating procedures (SOPs) for each major outcome domain including clinical, fluid biomarker, MRI, and digital-motor assessments (https://ataxia-global-initiative.net/working-groups/).

The goal of the AGI working group on COAs was to define a set of data including a graded catalog of COAs that will serve as the standard for future assessment and sharing of clinical data and joint clinical studies. The consensus was reached in one face-to-face meeting held during the 1st SCA Global Conference on 29 Mar 2019 in Las Vegas (NV, USA) followed by three virtual meetings of the working group. The final version of the recommendation was sent for approval by mail to the members of the working group. The working group was open to all AGI members. Formal consensus methods were not applied.

To keep the hurdles for the contribution of data to common analyses and studies low, it was agreed to define a mandatory dataset (minimal dataset) that can ideally be obtained during a routine clinical consultation and a more demanding extended dataset that is useful for research purposes. Additional information can be added for specific purposes. Data collection via phone or web meeting alone was not recommended.

### Minimal Dataset

The minimal dataset includes core data that provide basic information on demographics, clinical and genetic status, disability, and ataxia severity (Table 1). It includes the following items:

- Identifier: it is desirable to use a unique identifier with a large geographic reach, such as EUPID (https://eupid.eu).
- Participation in previous study/registry: if yes, the study acronym and (old) identifier should be entered.
- Core demographic data
- Genetic information: detailed genetic data according to common standards needs to be recorded. The query must be suitable for SCAs, recessive ataxias, and sporadic ataxias. Information on performed tests and negative results needs to be included. For this purpose, the genetic case report form (CRF) from autosomal recessive cerebellar ataxias (ARCA) registry can be used [8].
- Disability status: FARS functional staging for ataxia (FARS part I) [19] (http://www.ataxia-study-group.net/html/about/ataxiascales)
- Patient’s global impression of change (PGI) (7-point Likert scale related to functional impairment due to ataxia compared to the situation one year ago)
- SARA [15] (http://www.ataxia-study-group.net/html/about/ataxiascales)

**Table 1:** Minimal dataset

Item	Description	Justification
Identifier	Unique identifier, such as EUPID (https://eupid.eu)	Prevention of duplicate registration of patients Preservation of the possibility for re-identification by a trusted third party Facilitation of creating merged, datasets for secondary use (https://eupid.eu)
Participation in previous study/registry	Acronym of previous study/registry Identifier of participant	Facilitation of merging data from different studies/cohorts
Core demographic data	Sex, age, age at ataxia onset	Essential information for characterization of participants
Genetic information	Information on pathogenic and likely pathogenic genetic variant Information on performed tests and negative results	Essential information for stratification of participants
Disability status	Friedreich Ataxia Rating Scale (FARS) functional staging for ataxia (FARS part I)	Staging system providing a finer gradation compared to other systems
Patient’s global impression of change (PGI)	7-point Likert scale related to functional impairment due to ataxia compared to the situation one year ago	Highly relevant, patient-derived information on the disease course
Scale for the Rating and Assessment of Ataxia (SARA)	Scale for the Rating and Assessment of Ataxia (SARA)	SARA is the most widely used ataxia scale with a large amount of available data

### Extended Dataset

The extended set of COAs includes the following:

- Comorbidities
- As a standard, the comorbidity CRF from the ARCA registry [8] is recommended.
- Medication
- INAS [21] (http://www.ataxia-study-group.net/html/about/ataxiascales)
- FARS ADL (FARS part II) [19] (http://www.ataxia-study-group.net/html/about/ataxiascales)
- PROM-Ataxia [10]
- CCAS scale [23]

## Outlook

SARA is currently the most widely used clinical scale in the ataxia field. However, SARA is not generally considered usable for clinical trials. Specifically, patient relevance and clinical meaningfulness have not been systematically addressed. In addition, items differentially contribute to the SARA some score and have different sensitivity to change [32]. There are also practical problems with the application of single items resulting from multiple tasks, potentially contradictory criteria, and dependence on patient’s cooperation. It is therefore mandatory to systematically reassess SARA using existing data from natural history studies and interventional trials and to consider modifications based on the results of the reassessment. The goal should be to develop SARA into a generally accepted ClinRO that can be used in upcoming clinical trials.

PROs provide unique information on the impact of a disease from the patients’ perspective. Therefore, PROs should be included as endpoints in clinical trials to ensure that the impact of a trial intervention is comprehensively assessed. Indeed, PROs are increasingly used in trials, partly attributable to the top-down encouragement of regulatory bodies [33, 34]. PROM-ataxia is an ataxia-specific PRO that is considered a suitable instrument to assess the impact of ataxia on patients’ daily lives. However, data from homogeneous patient cohorts and longitudinal data are still lacking, and superiority compared to other ADL scales, such as the FARS-ADL, remains to be shown.

Systematic patient-derived data on meaningful aspects of health (MAH) are indispensable for anchoring the patient relevance and clinical meaningfulness of COAs, such as SARA, as well as of other outcome types [35]. Methods and evidence for anchoring COA change in patient meaningfulness are still missing for most COAs (e.g., by determining minimally meaningful thresholds of change anchored in patient-derived MAH). A procedure for this, however, has recently been proposed [36].

Finally, given the inherently small sample sizes accessible for almost all ataxias and the variability inherent in COAs, future work needs to focus on improving sensitivity to change of many COAs, including not only the SARA, but also the FARS-ADL, PROM-ataxia, and others.

## Data Availability

Not applicable.
